# Centromere transcription allows CENP-A to transit from chromatin association to stable incorporation

**DOI:** 10.1083/jcb.201611087

**Published:** 2018-06-04

**Authors:** Georg O.M. Bobkov, Nick Gilbert, Patrick Heun

**Affiliations:** 1 Wellcome Trust Centre for Cell Biology and Institute of Cell Biology, School of Biological Sciences, The University of Edinburgh, Edinburgh, Scotland, UK; 2 Faculty of Biology, Albert Ludwigs Universität Freiburg, Freiburg, Germany; 3 Medical Research Council Human Genetics Unit, Institute of Genetics and Molecular Medicine, The University of Edinburgh, Edinburgh, Scotland, UK

## Abstract

Centromeres are essential for chromosome segregation and are specified epigenetically by the presence of the histone H3 variant CENP-A. In flies and humans, replenishment of the centromeric mark is uncoupled from DNA replication and requires the removal of H3 “placeholder” nucleosomes. Although transcription at centromeres has been previously linked to the loading of new CENP-A, the underlying molecular mechanism remains poorly understood. Here, we used *Drosophila melanogaster* tissue culture cells to show that centromeric presence of actively transcribing RNA polymerase II temporally coincides with de novo deposition of dCENP-A. Using a newly developed dCENP-A loading system that is independent of acute transcription, we found that short inhibition of transcription impaired dCENP-A incorporation into chromatin. Interestingly, initial targeting of dCENP-A to centromeres was unaffected, revealing two stability states of newly loaded dCENP-A: a salt-sensitive association with the centromere and a salt-resistant chromatin-incorporated form. This suggests that transcription-mediated chromatin remodeling is required for the transition of dCENP-A to fully incorporated nucleosomes at the centromere.

## Abstract (precis)

How transcription contributes to the loading of the centromere histone CENP-A is unclear. Bobkov et al. report that transcription-mediated chromatin remodeling enables the transition of centromeric CENP-A from chromatin association to full nucleosome incorporation.

## Introduction

The centromere is a unique chromatin domain essential for proper segregation of chromosomes during mitosis. In most species, the position of the centromere is determined epigenetically by the specific incorporation of the histone H3-variant CENP-A (also called CID in *Drosophila melanogaster*; Earnshaw and Migeon, 1985; Karpen and Allshire, 1997; Henikoff et al., 2000). Although the presence of CENP-A is required to determine centromere identity, centromeric chromatin is composed of both CENP-A– and H3-containing nucleosomes that are arranged as interspersed domains (Blower et al., 2002; Bergmann et al., 2011; Martins et al., 2016). To act as a mark for the centromere, the replicative dilution of CENP-A during each S phase must be counteracted by cell cycle–coupled incorporation of new CENP-A. In *Drosophila*, new dCENP-A is incorporated into chromatin by its dedicated chaperone CAL1, which is recruited to the centromere via dCENP-C (Schittenhelm et al., 2010; Chen et al., 2014), and these three proteins have been found to be mutually interdependent for their centromeric localization (Erhardt et al., 2008). In contrast to canonical histone H3, which is replenished during S phase (Ahmad and Henikoff, 2002a,b), loading of CENP-A in humans and *Drosophila* takes place from mitosis to G1 (Jansen et al., 2007; Hemmerich et al., 2008; Dunleavy et al., 2012; Lidsky et al., 2013). Consequently, H3- and H3.3-containing “placeholder” nucleosomes are assembled at sites of CENP-A during replication of centromeric chromatin, which must be removed during the replication-independent loading of CENP-A (Dunleavy et al., 2011).

Over the last decade, active transcription has been recurrently linked to centromeres. Chromatin immunoprecipitation detected RNA polymerase II (RNAPII) at the central core domain of centromeres in *Schizosaccharomyces pombe* (Choi et al., 2011; Catania et al., 2015) and on human artificial chromosome (HAC) centromeres in human cells (Bergmann et al., 2011). Further analysis by immunofluorescence (IF) revealed the presence of RNAPII at endogenous centromeres on metaphase spreads of human (Chan et al., 2012) or fly (Rošić et al., 2014) cells and on stretched chromatin fibers of early G1 HeLa cells (Quénet and Dalal, 2014). Low-level transcription of centromeres is required for centromere function on endogenous centromeres in budding yeast (Ohkuni and Kitagawa, 2011) and on HACs, where transcriptional silencing resulted in a failure to load new CENP-A (Nakano et al., 2008; Cardinale et al., 2009; Bergmann et al., 2011). However, strong transcriptional up-regulation is also incompatible with centromere function, as it leads to rapid removal of CENP-A (Hill and Bloom, 1987; Bergmann et al., 2012). RNA transcripts derived from centromeric DNA have been reported in various organisms (Bergmann et al., 2011; Choi et al., 2011; Chan et al., 2012; Quénet and Dalal, 2014; Rošić et al., 2014; McNulty et al., 2017), and posttranslational modifications of histones that correlate with active transcription are present at centromeres (Sullivan and Karpen, 2004; Bergmann et al., 2011; Ohzeki et al., 2012).

In addition to generating RNA transcripts, transcription is accompanied by chromatin remodeling to allow regulated expression of genes and noncoding RNAs (Williams and Tyler, 2007). Fully assembled chromatin represents an obstacle for transcription and elongating polymerase complexes (Knezetic and Luse, 1986; Lorch et al., 1987; Izban and Luse, 1991), which is used by the cell to prevent general transcription of all DNA. The histone chaperone facilitates chromatin transcription (FACT) enables RNAPII to transcribe chromatinized DNA by destabilizing nucleosomes in front of the polymerase and reassembling them in its wake (LeRoy et al., 1998; Orphanides et al., 1998; Belotserkovskaya et al., 2003; Kaplan et al., 2003; Jamai et al., 2009; Morillo-Huesca et al., 2010). In vitro data further demonstrated that this transcription-induced destabilization can result in full eviction of nucleosomes by multiple, closely spaced transcribing RNAPII complexes (Kulaeva et al., 2010). Accordingly, transcribed regions of the genome show signs of elevated histone turnover, such as reduced nucleosome densities (Lee et al., 2004; Schwabish and Struhl, 2004) and increased levels of H3.3, which marks active chromatin by replication-independent nucleosome assembly (Ahmad and Henikoff, 2002b; McKittrick et al., 2004).

Interestingly, FACT was previously detected at centromeric chromatin (Foltz et al., 2006; Izuta et al., 2006; Okada et al., 2009; Chen et al., 2015; Prendergast et al., 2016) and has been linked to proper loading of new CENP-A. Although it prevents promiscuous misincorporation of CENP-A into noncentromeric locations in yeast (Choi et al., 2012; Deyter and Biggins, 2014), FACT is involved in the centromeric deposition of CENP-A in chicken (Okada et al., 2009) and flies (Chen et al., 2015). The involvement of FACT suggests a potential role of transcription-mediated chromatin remodeling in the CENP-A loading process (Choi et al., 2011; Chen et al., 2015), yet there is little understanding of how transcription contributes to this process at the molecular level.

In the present study, we treated *Drosophila* tissue culture cells for short periods with transcriptional inhibitors to analyze how transcription mechanistically affects the dCENP-A loading process. We found that RNAPII-mediated centromeric transcription and associated chromatin remodeling is required for dCENP-A to transit from an unstable chromatin-associated state to stably incorporated nucleosomes at the centromere.

## Results

### RNAPII localizes to centromeres in mitosis and G1

**Figure 1.**
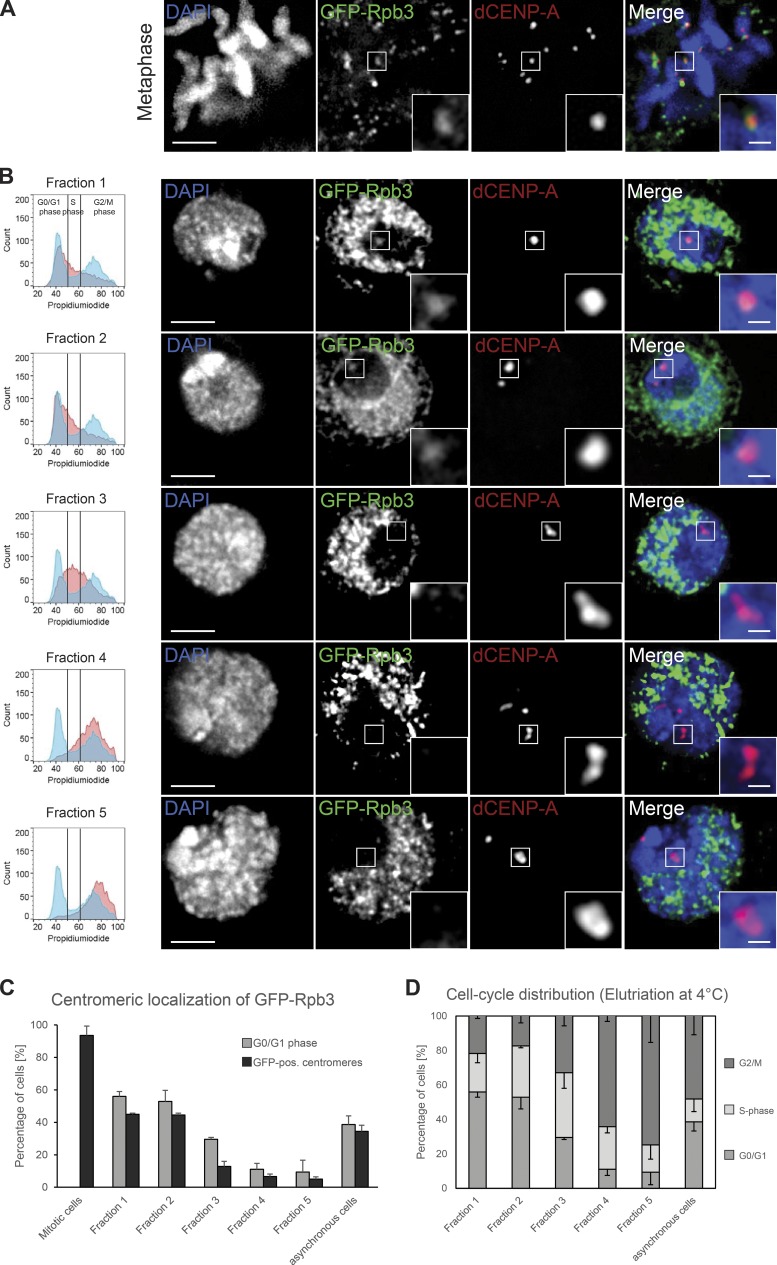
**Rpb3 localizes to centromeres in a cell cycle–restricted manner. (A and B)** Fixed S2 cells immunostained for dCENP-A as a marker for centromeres. Bar, 3 µm. Boxes indicate the 3× enlarged inset (bar, 0.5 µm). **(A)** Maximum-intensity projection of metaphase cell expressing GFP-Rpb3. Cells were prelysed in PBS/0.1% Triton X-100 for 30 s. **(B)** Single optical section of cells expressing GFP-Rpb3 elutriated into fractions to enrich for cells in G1 (fractions 1 and 2), S (fraction 3), and G2/M (fractions 4 and 5). The respective FACS profile for each fraction (red) in comparison to nonelutriated cells (blue) is shown in the left column. **(C)** Graph displaying the correlation between the amount of G1 cells and the cells that showed GFP-positive centromeres in all elutriation fractions, mitotic cells, and asynchronous growing cultures. *n* = 3 replicates; *n* = 30–130 cells; data are mean + SD. **(D)** Graph depicting the presence of the various cell populations in the elutriation fractions and asynchronous growing cultures. Elutriation was performed at 4°C; data were extracted from FACS profiles for each fraction (see also left column of B). *n* = 3. Data are represented as mean − SD.

To perform a detailed localization analysis of *Drosophila* RNAPII throughout the cell cycle, we used an N-terminal GFP-fusion of its subunit Rpb3. As expected, GFP-Rpb3 localized to centromeres in metaphase (Fig. 1 A) and was displaced from other regions of mitotic chromosomes, as genome transcription is mostly silenced during mitosis (Gottesfeld and Forbes, 1997). In addition, GFP-Rpb3 remained associated with centromeres in later mitotic stages (Fig. S1 A) and is detectable at centromeres in early G1 cells (Fig. S1 B), together with broad euchromatin staining in the rest of the genome. In contrast, the heterochromatic areas that surround the centromere were largely devoid of any signal. Signal intensities of centromeric GFP-Rpb3 between interphase and mitotic cells were similar (Fig. S1 C), indicating that centromeres are transcribed in both mitosis and a subset of interphase cells. To map the interphase centromeric localization of RNAPII in more detail, we investigated GFP-Rpb3 localization in elutriated cells. In an elutriation centrifuge, cells can be separated according to their size by gradually increasing the force created by the flow of the cell-containing medium, which opposes the centrifugal force created by the spinning rotor. This allowed us to enrich for small G1-phase (fractions 1 and 2), medium-sized S-phase (fraction 3), and large G2/M-phase (fractions 4 and 5) cells, respectively (Fig. 1 B, left column). Subsequent microscopy analysis of interphase cells revealed that GFP-Rpb3–positive centromeres were mostly detected in cells of fractions 1 and 2, whereas centromeres of cells in fractions 4 and 5 were largely depleted of a GFP signal (Fig. 1, B and C). Low levels of GFP-positive centromeres in fractions that are mostly comprised of S- and G2/M-phase cells (fractions 3–5) directly correlate with the amount of contaminating G1 cells in each fraction (Fig. 1, C and D).

### Centromere-associated transcripts temporally coincide with dCENP-A loading

**Figure 2.**
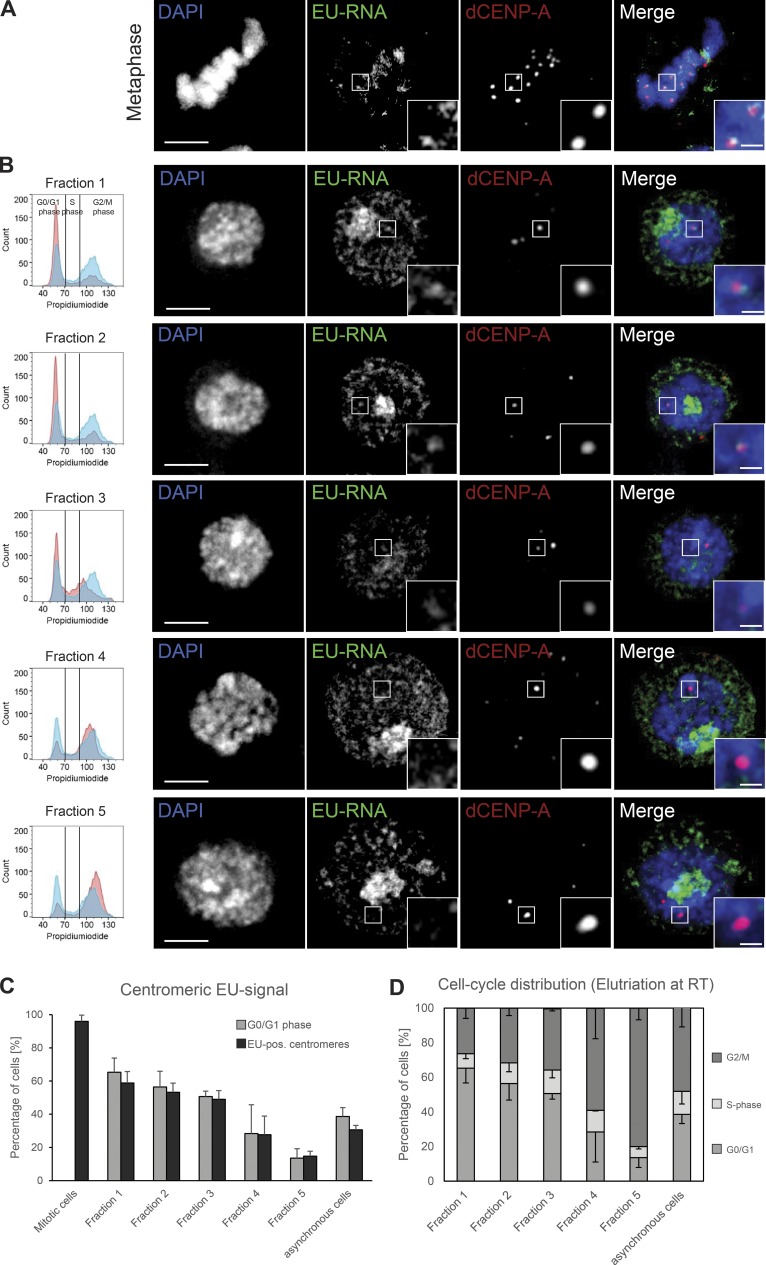
**Nascent RNA transcripts are present at mitotic and interphase centromeres. (A and B)** Fixed S2 cells immunostained for dCENP-A as a marker for centromeres. Bar, 3 µm. Boxes indicate the 3× enlarged inset (bar, 0.5 µm). **(A)** Maximum-intensity projection of metaphase cell with nascent RNA production labeled by EU incorporation. **(B)** Single optical section of cells elutriated into fractions to enrich for cells in G1 (fractions 1–3) and G2/M (fractions 4 and 5). Nascent RNA production was labeled by EU incorporation. The respective FACS profile for each fraction (red) in comparison to nonelutriated cells (blue) is shown in the left column. **(C)** Graph displaying the correlation between the amount of G1 cells and the cells that showed EU-RNA–positive centromeres in all elutriation fractions, mitotic cells, and asynchronous growing cultures. *n* = 3 replicates; *n* = 30–100 cells; data are mean + SD. **(D)** Graph depicting the presence of the various cell populations in the elutriation fractions and asynchronous growing cultures. Elutriation was performed at RT; data were extracted from FACS profiles for each fraction (see also left column of B). *n* = 3 replicates; data are mean − SD.

The localization of a subunit of RNAPII is an indication, but not proof, of active transcription at mitotic and interphase centromeres. To visualize potential centromere-associated transcripts, we labeled nascent RNA using the Click-iT technology. In this method, a 5-ethynyl uridine substrate (EU) is actively incorporated during RNA synthesis and can subsequently be labeled by ligation to a fluorescent dye. Short pulse-labeling of asynchronously growing S2 cells with EU revealed nascent RNA transcripts associated with mitotic centromeres (Fig. 2 A), indicating that the centromeric RNAPII is actively transcribing. Although newly synthesized RNA in interphase was largely dominated by strong RNA production at the ribosomal DNA locus in the nucleolus, nascent transcripts emanating in the vicinity of centromeres were detectable in interphase cells as well (Fig. 2 B, upper panel; and Fig. S1 D, non–EU-control).

To define the specific cell cycle phases in which centromeric transcripts are produced, we labeled nascent RNA in freshly elutriated cells. This required the elutriation to be performed at RT, which resulted in a depletion of S-phase cells from all obtained fractions (compare Figs. 1 D and 2 D). In agreement with the cell cycle–regulated centromeric localization of RNAPII (Fig. 1), the percentage of cells showing nascent centromeric transcripts directly correlated with the amount of G1 cells in each fraction (Fig. 2, B and C). To clarify whether nascent centromeric transcripts are produced in S-phase cells, we costained EU-labeled cells with the DNA replication marker proliferating cell nuclear antigen (PCNA) and found that centromeric transcripts were absent in both early and late S-phase cells (Fig. S1 E).

Collectively, these data show that the presence of RNAPII and the production of nascent RNA transcripts at the centromere start in mitosis and end in G1 phase. Intriguingly, this cell cycle window correlates with the incorporation of new dCENP-A into *Drosophila* centromeres (Dunleavy et al., 2012; Lidsky et al., 2013).

### A transcription-independent dCENP-A loading system

**Figure 3.**
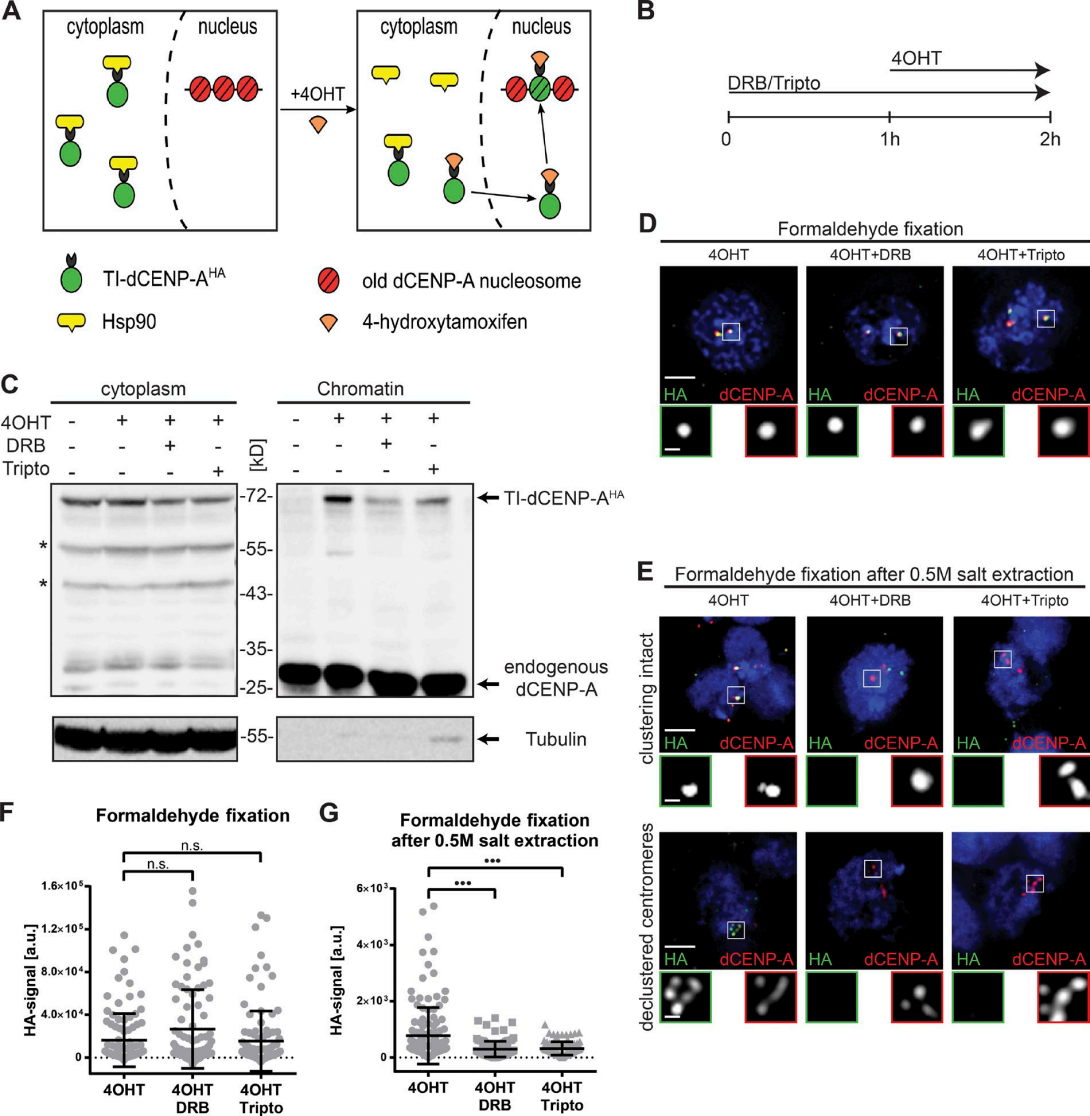
**Transcriptional inhibition prevents stabilization of correctly targeted new dCENP-A. (A)** Schematic illustration of the tamoxifen-mediated release of TI-dCENP-A^HA^. **(B)** Experimental setup used in C–G and Figs. 4, 5 (D and E), 6, S3, and S4 (B–D). **(C)** Western blot analysis showing the impaired loading of new dCENP-A into chromatin after transcriptional inhibition. Arrows mark protein of interest, and asterisks mark unspecific bands or potential degradation products. Endogenous dCENP-A (chromatin) and tubulin (cytoplasm) serve as markers for the two fractions. **(D)** Maximum-intensity projection of cells stably expressing TI-dCENP-A^HA^ fixed in formaldehyde. Respective treatments are indicated above each picture. Bar, 3 µm. 3× magnification of boxed area is shown below (TI-dCENP-A^HA^ [green] and total dCENP-A [red]; bar, 0.5 µm). **(E)** Maximum-intensity projection of cells stably expressing TI-dCENP-A^HA^ fixed in formaldehyde after 30 min of 0.5 M salt extraction. Cells with clustering of centromeres still intact (upper panel) and disrupted (lower panel) are shown. Respective treatments are indicated above the pictures. Bars, 3 µm. 3× magnification of boxed area is shown below (TI-dCENP-A^HA^ [green] and total dCENP-A [red]; bar, 0.5 µm). **(F and G)** Quantification of centromeric HA-signal/cell from pictures shown in D (F) or E (G). *n* = 3 replicates, *n* = 30–50 cells. Data are mean ± SD. The p-value was determined using the Kolmogorov–Smirnov test. •••, P ≤ 0.001.

Based on the cell cycle analysis described above, we decided to test how an acute block of transcription affects de novo incorporation of dCENP-A. To distinguish between old and new proteins, previous experimental approaches relied on transcription to produce new proteins after either a labeling event (SNAP-tag; Jansen et al., 2007) or a recombination event (recombination-induced tag exchange; Verzijlbergen et al., 2010) and are thus not compatible with concurrent transcriptional inhibition. To overcome this problem, we developed an experimental system that provides new dCENP-A independently of acute gene transcription. Adopting the well-established inducible tamoxifen system (Feil et al., 1996; Indra et al., 2005), we fused dCENP-A to an HA-tagged estrogen receptor variant. Because of an interaction with Hsp90, the resulting fusion protein is retained in the cytoplasm and released into the nucleus only upon addition of 4-hydroxytamoxifen (4OHT; Fig. 3 A). We refer to this construct as tamoxifen-inducible HA-tagged dCENP-A (TI-dCENP-A^HA^).

Cellular fractionation of cells stably expressing TI-dCENP-A^HA^ showed that 4OHT treatment was required for its detection in the chromatin fraction (Fig. S2 A). IF analysis further revealed that its specific localization to centromeres depended on 4OHT (Fig. S2 B), and, as previously reported, HA-positive centromeres were detectable already in metaphase cells (Fig. S2 C; Mellone et al., 2011). Once released, live imaging of cells demonstrated that TI-dCENP-A^GFP^ is incorporated into centromeres in a manner identical to newly transfected dCENP-A^mCherry^ (Fig. S2, D and E). We observed no loading in G2, followed by a transient peak of TI-dCENP-A^GFP^ loading in mitosis and a slow increase in the centromeric signal during the subsequent G1 phase. This confirms that TI-dCENP-A^HA^ behaves like normal dCENP-A upon 4OHT addition but is unable to contribute to dCENP-A assembly before its release (Fig. S2, A, B, and F). The fact that already ∼42% of all cells reacted after 1 h of 4OHT treatment further supports that both mitosis and most of G1 are permissive to dCENP-A loading, whereas the comparably small increase to ∼55% after 4 h of treatment can be attributed to cells newly entering this cell cycle window (Fig. S2 F).

### RNAPII transcription promotes stabilization of new dCENP-A at centromeres

To study the effect of RNAPII inhibition on the incorporation of TI-dCENP-A^HA^ into centromeres, we chose two fast-acting (minutes) inhibitors of RNAPII for our experiments (Bensaude, 2011). Western blot analysis of total cell extracts confirmed that both triptolide (tripto) and 5,6-dichloro-1-β-d-ribofuranosylbenzimidazole (DRB) are effective, as phosphorylation markers indicative of active RNAPII (serine 2 and serine 5) were strongly reduced (Figs. S3 A). 2-h treatment of both inhibitors also led to a strong reduction of nascent RNA transcripts associated with centromeres in dumbbell-shaped early G1 cells (Fig. S3, B and C). Although tripto-treated cells also showed significant reduction for nascent RNA signals protruding from the nucleolus, this staining was not affected by the DRB inhibitor (Fig. S3 D). Specific inhibition of RNAPII by DRB and general inhibition of all three RNA polymerases by tripto was further confirmed by quantitative PCR (qPCR) on purified nascent RNAs (Fig. S3 E). Collectively, these findings strongly suggest that nascent RNA transcripts at the centromere are produced by RNAPII.

Next, we analyzed the progression of control and inhibitor-treated cells through mitosis, to exclude that a potential dCENP-A loading phenotype is caused by altered cell cycle progression. Live imaging of cells confirmed that the mitotic index of cell cultures was unchanged for inhibitor treatments of up to 2 h, whereas longer drug exposure started to block the passage of cells through mitosis and thus dCENP-A loading (Fig. S3 F). We therefore decided to block transcription for 2 h and combine it with a 1-h-lasting release of TI-dCENP-A^HA^ triggered by addition of 4OHT (Fig. 3 B).

Combining the newly developed dCENP-A loading system with short inhibition of RNAPII, chromatin fractionations of differentially treated cells were analyzed for the presence of TI-dCENP-A^HA^ in chromatin by Western blotting. Indeed, we found that de novo TI-dCENP-A^HA^ loading was compromised when transcription was simultaneously inhibited (Fig. 3 C). Interestingly, microscopy analysis of released TI-dCENP-A^HA^ revealed that inhibitor treatment had no effect on the localization of TI-dCENP-A^HA^ to centromeres when cells were fixed using standard PFA protocols (Fig. 3, D and F). Combined, these results suggest that an initial recruitment step of new dCENP-A to centromeres occurs independently of transcription, but that loading cannot be completed if transcription is inhibited. This interpretation is further strengthened by the localization behavior of released TI-dCENP-A^HA^ in IF of cells that were prepared using harsher fixation protocols. TI-dCENP-A^HA^ failed to remain at centromeres in inhibitor treated cells if chromatin-associated proteins were extracted by high salt before PFA fixation (500 mM NaCl, similar to cellular fractionation; Fig. 3, E and G). Prelysis of cells in high salt before fixation strongly affects cell morphology and can differ largely between cells on the same slide. We distinguished three groups with increased severity of extraction: cells in which centromere clustering remained intact (Fig. 3 E, upper panel); cells with declustered centromeres (Fig. 3 E, lower panel); and cells with largely destroyed nuclear integrity and visible chromatin fiber-like structures (Fig. S4 A). In each of these groups, new TI-dCENP-A^HA^ was only able to withstand the extraction forces when transcription was not simultaneously inhibited (Fig. 3, E and G). Likewise, centromeric TI-dCENP-A^HA^ levels were significantly reduced in inhibited cells fixed in methanol (Fig. S4, B and C), which has been previously used to differentiate the stability state of PCNA (Bravo and Macdonald-Bravo, 1987). Collectively, these results suggest that transcriptional inhibition prevents the transition of newly recruited and centromere-associated dCENP-A to a more stable state, as expected for a CENP-A nucleosome.

### Transcription is required for incorporation of new dCENP-A into chromatin

**Figure 4.**
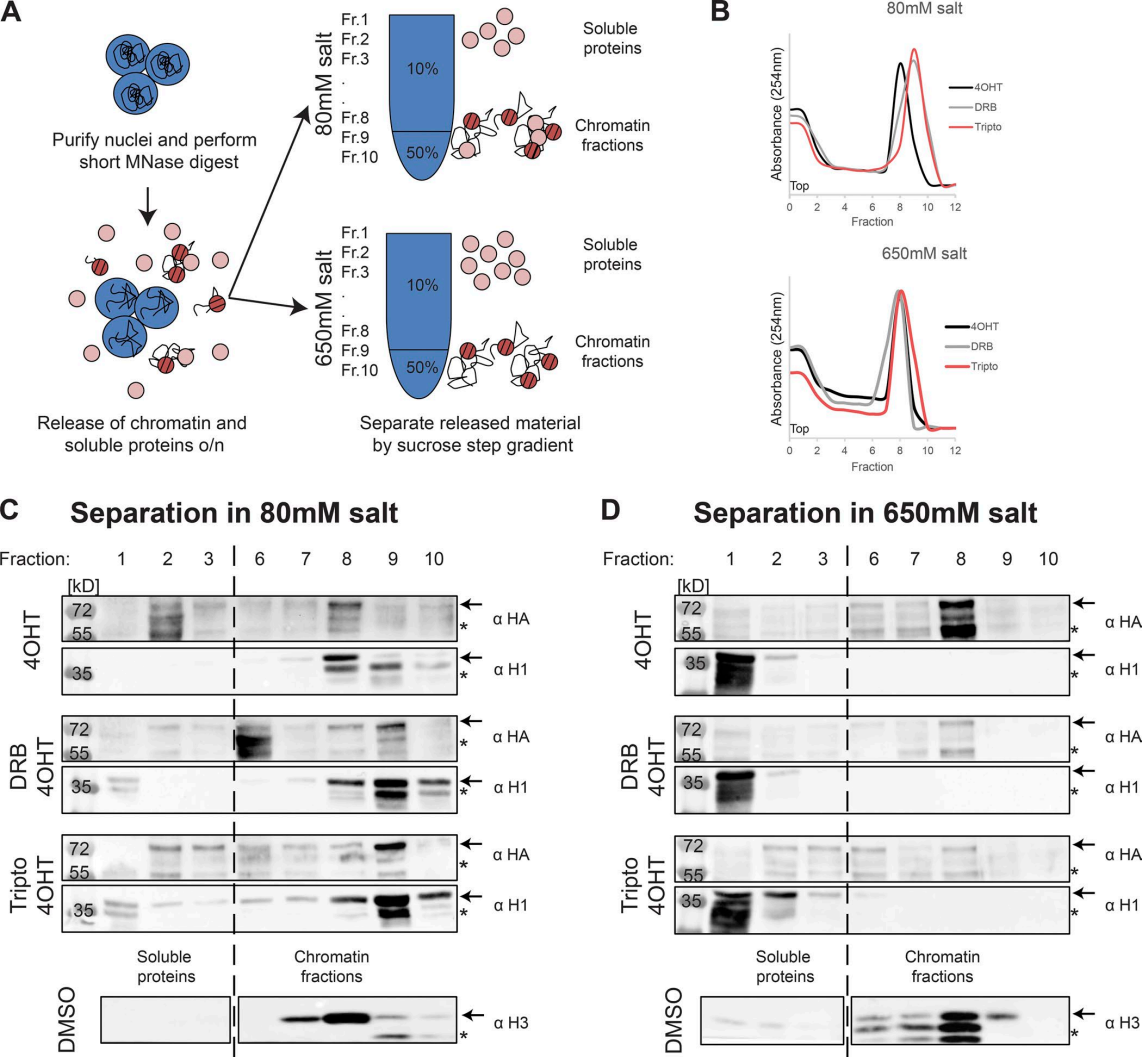
**Transcription is required for full incorporation of new dCENP-A. (A)** Experimental setup for chromatin fractionations on high- and low-salt sucrose step gradients analyzed in C and D. Fractions 1–3 contain soluble proteins; fractions 8–10 represent the chromatin fractions. o/n, overnight. **(B)** Chromatin of differentially treated cells stably expressing TI-dCENP-A^HA^ fractionated on a 10/50% sucrose step gradient. Chromatin was monitored by absorbance at 254 nm. **(C and D)** Western blot after separation on low-salt (C) or high-salt (D) step gradients. Treatment of sample is indicated on the left, and each sample was probed for TI-dCENP-A^HA^ (upper/α HA) and H1 (lower/α H1). Behavior of histone H3 is depicted for control treatment at the bottom. Arrows mark protein of interest and asterisks mark unspecific bands or potential degradation products.

It is intriguing to speculate that the two observed stability states of new dCENP-A represent a correctly targeted, but only loosely associated, pool versus stable, fully incorporated dCENP-A nucleosomes. To test this, we analyzed new TI-dCENP-A^HA^ from MNase-digested native chromatin by separating soluble proteins from chromatin proteins on a high-salt (650 mM NaCl) or low-salt (80 mM NaCl) sucrose step gradient (Fig. 4 A). Previous work has shown that nucleosomal CENP-A can withstand high-salt extractions (Ando et al., 2002), whereas chromatin-associated factors are removed from chromatin under these conditions. Independently of sample treatment or salt concentration of the sucrose step gradient, MNase-digested chromatin ran in fractions 8–10 as revealed by maximal DNA absorption at 254 nm (Fig. 4 B) and presence of histone H3 (Fig. 4, C and D). Accordingly, the chromatin-associated histone H1 was present in fractions 8–10 (chromatin) after separation on the low-salt column but was released into fractions belonging to soluble proteins (fractions 1–3) when loaded onto a high-salt gradient (Fig. 4, C and D). Unlike the clear band visible for H1 in its chromatin-associated state (Fig. 4 C), a smear of partial degradation products was detected for H1 released from chromatin by high salt, indicating increased degradation of salt-released soluble proteins during the experimental procedure (Fig. 4 D). Fully incorporated histone H3, which served as a control for incorporated nucleosomes, largely withstood the extraction forces posed by 650 mM NaCl and was always present in chromatin fractions 8–10 (Fig. 4, C and D). As expected, 4OHT-released TI-dCENP-A^HA^ in transcriptionally active cells behaved like H3 and remained in the chromatin fractions irrespective of salt concentrations, indicating its full incorporation into chromatin. In contrast, TI-dCENP-A^HA^ was largely removed from chromatin fractions in both inhibitor-treated samples under high-salt conditions, suggesting a nonnucleosomal, salt-sensitive association with chromatin (Fig. 4, C and D). Unlike for H1, we could not observe the reappearance of a TI-dCENP-A^HA^ signal in the soluble protein fractions under high-salt conditions (Fig. 4 D). The reasons for this are unclear but likely involve degradation of salt-released proteins during the course of the experiment. This is supported by the fact that our chromatin sample was equally split between the low- and high-salt sucrose step gradients (Fig. 4 A), and chromatin-associated TI-dCENP-A^HA^ could clearly be detected in chromatin fractions under low-salt conditions (Fig. 4 C).

We conclude that the salt-sensitive pool of TI-dCENP-A^HA^ observed in the IF studies indeed represents nonincorporated dCENP-A, suggesting that transcription is required for the transition of merely associated dCENP-A to fully incorporated dCENP-A nucleosomes.

### Centromere-associated Sat III transcripts stem from local transcription

Previously, a long noncoding RNA transcript that originates from the 359-bp repeat satellite III (SAT III) of the X-chromosome has been described to act in trans at most centromeres, where it stabilizes dCENP-C and is generally required for dCENP-A and dCENP-C loading (Rošić et al., 2014).

**Figure 5.**
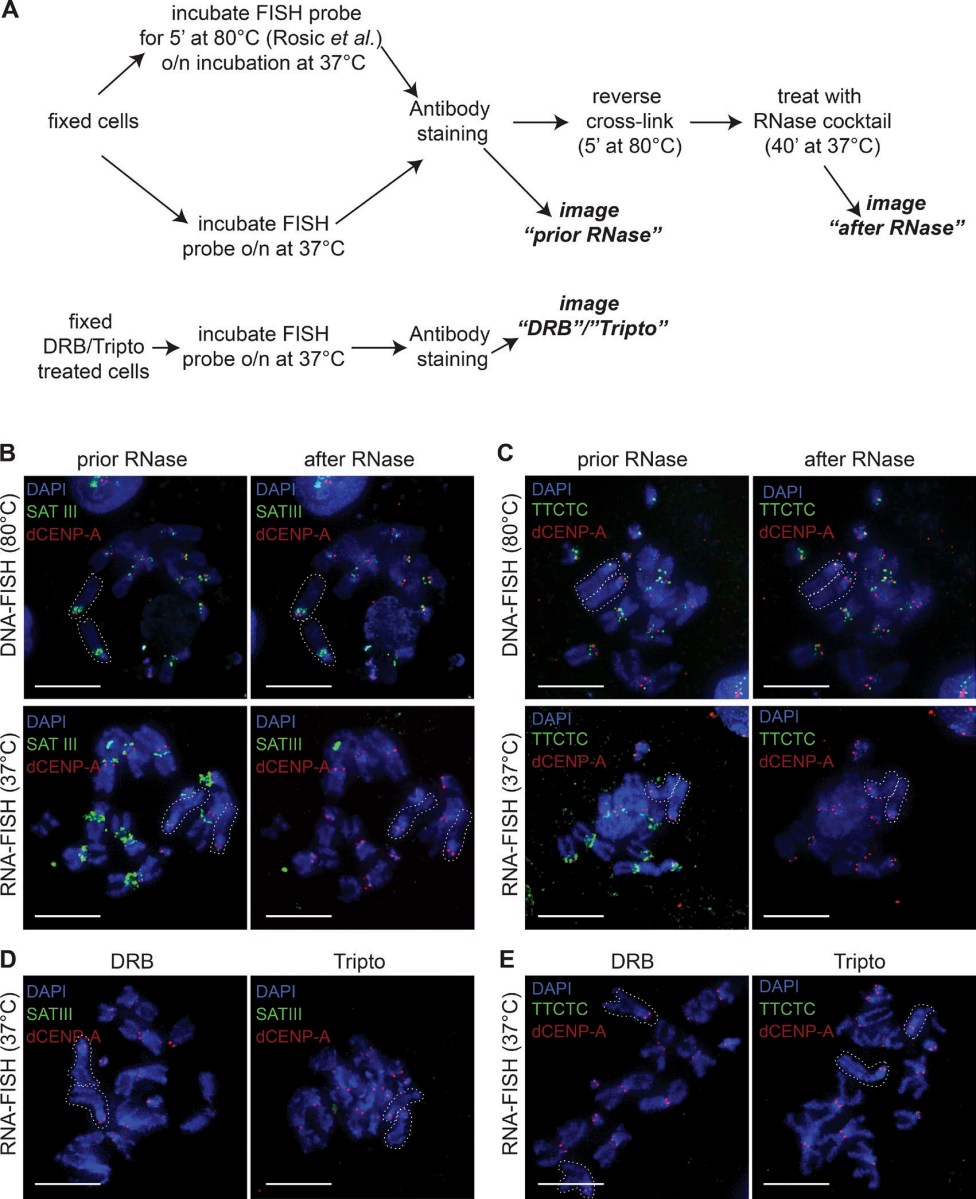
**Centromeric RNA transcripts in mitosis originate from local RNAPII transcription. (A)** Schematic illustration of the different FISH protocols used to differentiate between DNA-FISH (including DNA denaturation at 80°C) and RNA-FISH. o/n, overnight. **(B and C)** Identical metaphase spread labeled with FISH probes for SAT III (B) or TTCTC (C) were imaged before and after RNase treatment. DNA-FISH signals (upper panel) and RNA-FISH signals (lower panel) are shown. White dotted lines indicate the acrocentric X-chromosomes. **(D and E)** Metaphase spreads labeled with FISH probes for SAT III (D) or TTCTC (E) after 2-h treatment with DRB (left) or tripto (right) are shown. White dotted lines indicate the acrocentric X-chromosomes. Bars, 3 µm.

To test how our transcriptional inhibition affects this particular RNA transcript, we performed FISH experiments as described previously (Rošić et al., 2014; Fig. 5 A). We confirmed the presence of a FISH signal on the centromeres of the X-chromosome and the two major autosomes using a probe directed against the SAT III sequence. However, we found that the SAT III staining was not removed after RNase treatment (Fig. 5 B, upper panel), indicating that the observed signal represents genomic DNA. In contrast, when probe hybridization was completely performed at 37°C (RNA-FISH; Fig. 5 A), we could detect RNase-sensitive FISH signals for SAT III (Fig. 5 B, lower panel). Similar results were obtained with a control FISH probe against a simple TTCTC repeat (Fig. 5 C), originally identified on X-chromosome–derived minichromosomes (Sun et al., 2003) and found on centromeric and pericentromeric regions of all chromosomes (unpublished data). RNA- and DNA-FISH mark the same chromosomal regions (Fig. 5, B and C), and RNA signals for either probe disappear in cells pretreated with DRB or tripto (Fig. 5, D and E).

Together, these results suggest that centromeric SAT III RNA transcripts are produced in mitosis by RNAPII but stem from local RNA transcription of centromeric DNA sequences rather than acting in trans from the X-chromosome.

### Chromatin association of CAL1 is similar to new dCENP-A in inhibitor-treated cells

**Figure 6.**
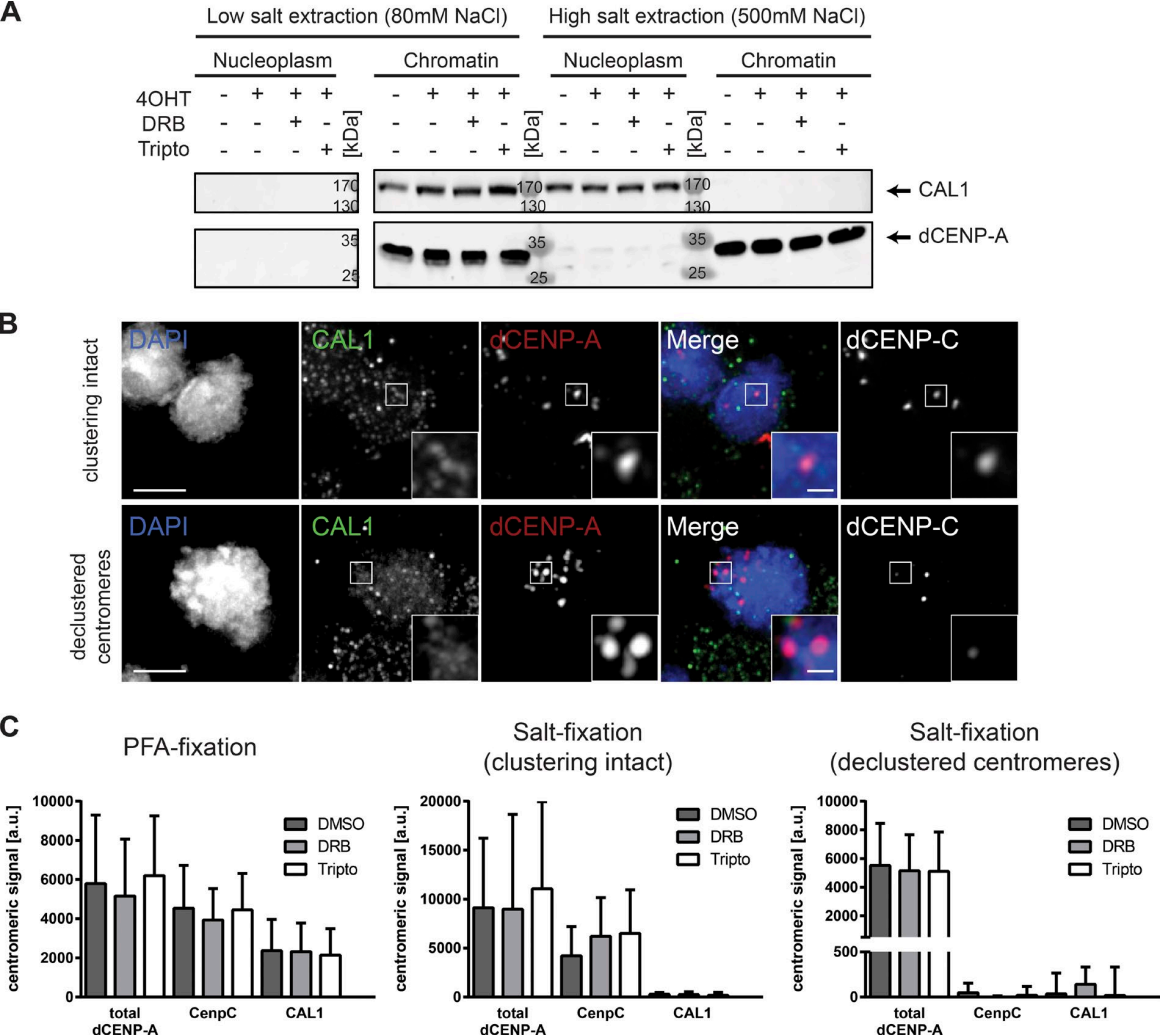
**Centromere association of CAL1 is less stable than CENP-C, and neither CAL1 nor CENP-C is sensitive to transcriptional inhibition. (A)** Western blot analysis showing the displacement of CAL1 from chromatin after high-salt extraction. Arrows mark protein of interest. Endogenous dCENP-A serves as a marker for chromatin. **(B)** Maximum-intensity projection of cells fixed after 30 min of 0.5 M salt extraction and immunostained for CAL1 and dCENP-C. Extracted cells with clustering of centromeres still intact (upper panel) and disrupted (lower panel) are shown. Bars, 3 µm. **(C)** Quantifications of centromeric localization of total dCENP-A, dCENP-C, and CAL1 in inhibitor-treated cells. Fixation type and group of analyzed cells is indicated above. *n* = 3 replicates; *n* = 25–50 cells. Data are mean + SD.

We next investigated the behavior of CAL1 and dCENP-C in inhibitor-treated cells, as both proteins are essential for proper localization of dCENP-A (Erhardt et al., 2008). In salt fractionation experiments, we detected CAL1 in chromatin fractions after low-salt extraction (80 mM NaCl), but it was present in the nucleoplasm after fractionation under high-salt conditions (500 mM NaCl; Fig. 6 A). Low stability of dCENP-C in cellular lysates prevented the analysis of this factor in these experimental conditions. However, analysis of both dCENP-C and CAL1 was possible in cells fixed in PFA with and without preextraction of associated proteins using high salt. Similar to the results obtained in the fractionation experiments, CAL1 localized to centromeres in PFA-fixed cells but was in all cells removed from centromeres by high-salt extraction before PFA fixation (Fig. 6, B and C). In contrast, dCENP-C remained associated with centromeres in salt-extracted cells, where centromere clustering is still intact, yet was displaced from declustered centromeres (Fig. 6, B and C). This is in agreement with previous findings made by the Karpen (Mellone et al., 2011) and Lehner (Lidsky et al., 2013) laboratories that found dCENP-C to be more stably associated with chromatin than CAL1. Importantly, neither the localization nor the protein levels of CAL1 and dCENP-C were affected by DRB or tripto treatment (Figs. 6 C and S4 D).

**Figure 7.**
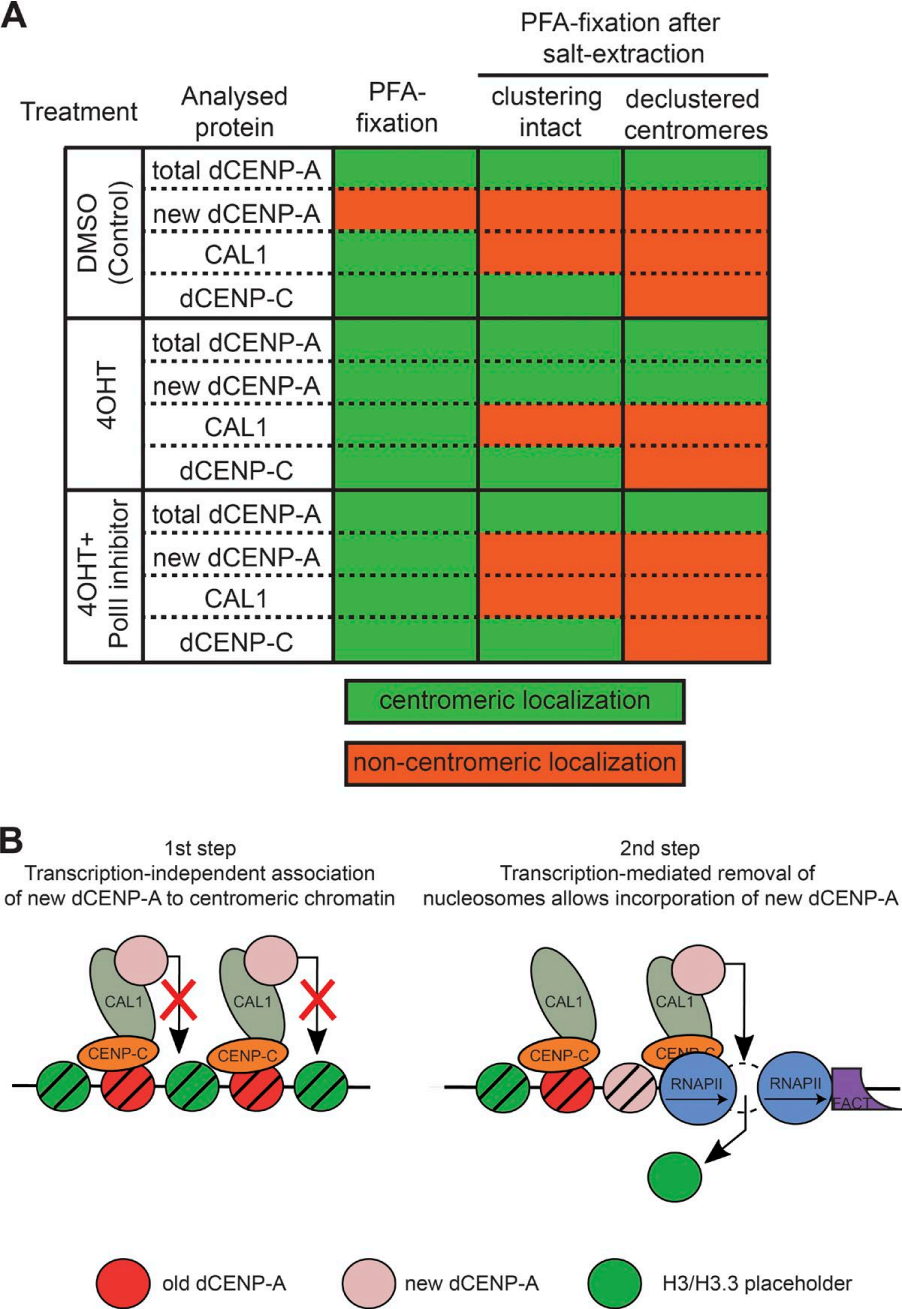
**Two-step model for assembly of new dCENP-A at centromeres. (A)** Table summarizing the data concerning the centromeric localization of proteins tested in Figs. 3 and 6. **(B)** Putative model for the assembly of new dCENP-A at centromere: (1) CAL1-targeted new dCENP-A/H4 associates with centromeres, but incorporation is blocked by previously incorporated placeholder nucleosomes; and (2) FACT-mediated transcription induces the eviction of nucleosomes, thus allowing the full incorporation of new dCENP-A.

In summary, dCENP-C is more stably associated with centromeres than CAL1, which in turn behaves identical to the unstable, only chromatin-associated TI-dCENP-A^HA^ in the inhibitor treated samples (Fig. 7 A).

## Discussion

Several studies have previously linked centromeric transcription to centromere function and de novo loading of CENP-A (Okada et al., 2009; Chan et al., 2012; Quénet and Dalal, 2014; Rošić et al., 2014; Grenfell et al., 2016). Recently, it has been shown that long-term interference with centromeric transcription by depletion of FACT results in reduced centromeric dCENP-A levels (Chen et al., 2015). However, the immediate contribution of transcription in this process remains poorly understood. This prompted us to use the *Drosophila* model system to investigate how dCENP-A deposition is affected by inhibition of acute transcription.

Using a GFP-tagged subunit of RNAPII, we found that RNAPII associates with centromeres from metaphase to G1, but not in S and G2. This centromeric RNAPII is transcriptionally active, as nascent centromere-associated transcripts disappear in cells treated with RNAPII inhibitors. Because this cell cycle period matches the time window for new dCENP-A deposition in fly cells (Dunleavy et al., 2012; Lidsky et al., 2013), we decided to rigorously test the effect of transcriptional inhibition on de novo incorporation of dCENP-A in Schneider S2 cells. To this end, we developed a system to assess the incorporation of new proteins independently of acute transcription, using tamoxifen-induced release of ready-made TI-dCENP-A^HA^. Indeed, we found RNAPII transcription to be important for dCENP-A incorporation, as reduced levels of TI-dCENP-A^HA^ were detected in the chromatin fraction of inhibitor-treated cells. Interestingly, IF microscopy analysis revealed that recruitment of new dCENP-A to centromeres was unaffected by RNAPII inhibition. This apparent paradox was solved using harsher fixation conditions, such as high-salt preextraction (similar to the conditions used to prepare the chromatin fraction) or methanol fixation, in the preparation of the microscopy samples. Although TI-dCENP-A^HA^ remained at centromeres even under harsher fixation conditions in transcriptionally active cells, it was strongly reduced in DRB- or tripto-treated cells. Together, these results suggest that new dCENP-A can localize properly to centromeres in inhibitor-treated cells, but stable incorporation into chromatin is compromised.

The stability of dCENP-A was characterized in more detail by analyzing chromatin fragments on sucrose step gradients containing high or low salt. When released without RNAPII inhibition, TI-dCENP-A^HA^ behaved like fully incorporated histone H3, whereas in inhibitor-treated samples its distribution was similar to chromatin-associated histone H1. We conclude that new centromeric dCENP-A exists in two distinct stability states that are separated by transcription: first, new dCENP-A associates with centromeres in a transcription-independent manner and is only fully incorporated in nucleosomes in a second, transcription-dependent step (Fig. 7 B). This finding is particularly interesting in light of an earlier study in HeLa cells, in which the knockdown of the chromatin remodeler Rsf1 also led to salt-sensitive centromere association of newly loaded CENP-A (Perpelescu et al., 2009). As chromatin remodelers are generally involved in transcription of chromatinized templates (LeRoy et al., 1998), a cooperative mechanism is plausible.

The replenishment of the centromeric mark requires large-scale chromatin remodeling, as previously incorporated placeholder nucleosomes need to be exchanged against CENP-A–containing ones (Dunleavy et al., 2011). Intriguingly, transcription has the ability to evict nucleosomes from chromatin (Kristjuhan and Svejstrup, 2004; Lee et al., 2004; Schwabish and Struhl, 2004; Daury et al., 2006; Kulaeva et al., 2010), which triggers the deposition of nucleosomes containing the H3 variant H3.3 in transcribed regions of the genome (Ahmad and Henikoff, 2002b). Because we found that inhibition of acute transcription prevents the incorporation of dCENP-A despite its proper recruitment to centromeres, it is tempting to speculate that transcription of centromeres is required to induce the eviction of placeholder nucleosomes (Choi et al., 2011; Chen et al., 2015). In such a model, dCENP-A incorporation would occur in a gap-filling mode similar to that observed for H3.3 in other regions of the genome (Fig. 7 B). Indeed, the induction of histone turnover events on an ectopic array of α-satellite DNA can result in both H3.3 and dCENP-A deposition (Shono et al., 2015; Ohzeki et al., 2016), and misincorporation of overexpressed dCENP-A (Heun et al., 2006; Olszak et al., 2011) mimics the general distribution pattern of H3.3 (Ahmad and Henikoff, 2002b; Wirbelauer et al., 2005). In addition, CENP-A incorporation into noncentromeric regions is elevated in experimental conditions that increase the appearance of nucleosomal gaps in chromatin (Au et al., 2008; Lopes da Rosa et al., 2011; Choi et al., 2012). Interestingly, recent work from the Earnshaw laboratory (Molina et al., 2016) demonstrated that the failure to load new CENP-A at transcriptionally silenced centromeres of HACs can be rescued by simultaneous deposition of H3K9 acetylation (H3K9ac), but not H4K12ac. Although both modifications restored HAC centromere transcription, CENP-A incorporation and importantly also the removal of placeholder nucleosomes were only achieved if transcription was reintroduced by deposition of H3K9ac (Molina et al., 2016). As H3ac but not H4ac has been shown to destabilize nucleosomes (Gansen et al., 2015), this further supports a model where a crucial contribution of centromeric transcription in the CENP-A loading process lies in the destabilization and eviction of placeholder nucleosomes.

With CAL1, dCENP-C, and the long noncoding SAT III transcript, three different factors have previously been described to affect the localization of *Drosophila* dCENP-A directly (Erhardt et al., 2008; Rošić et al., 2014). To clarify their roles in dCENP-A loading with respect to transcriptional inhibition, we tested all three components for changes in their localization in RNAPII inhibitor–treated cells. The SAT III transcript has previously been described to be produced exclusively at the proximal heterochromatic DNA of the X-chromosome. These transcripts were reported to localize in trans to the centromeres of all major chromosomes in S2 cells, where they promote dCENP-C stability and dCENP-A loading (Rošić et al., 2014). Here, we present evidence that the observed SAT III FISH signals represent genomic DNA sequences rather than RNA transcripts based on the FISH protocol used, which includes a DNA denaturation step at 80°C (typically used for DNA-FISH protocols) and insensitivity of the probe signal to RNase treatment. The additional presence of SAT III DNA on the second and third centromere is also in line with the original mapping of the repeat to *Drosophila* chromosomes (Lohe et al., 1993). The nature of the respective FISH signals was independently confirmed through similar treatment of a control probe directed against a previously identified centromeric DNA sequence, the simple TTCTC repeat (AAGAG; Lohe et al., 1993; Sun et al., 1997). However, using an RNA-FISH protocol omitting the DNA denaturation step, we found that both SAT III DNA and the TTCTC DNA were transcribed during mitosis. Both RNA transcripts can be detected on metaphase spreads, are sensitive to RNase treatment, and are strongly reduced by RNAPII inhibitor treatment. Therefore, the most direct interpretation of our results is that centromeric SAT III RNAs are produced locally by RNAPII rather than populating other centromeres in trans. This is also in agreement with recent findings that transcripts involved in CENP-A loading are produced in cis at human centromeres (McNulty et al., 2017).

The dCENP-A dedicated chaperone CAL1 and dCENP-C are both essential for the proper localization of dCENP-A to centromeres (Erhardt et al., 2008). We found that dCENP-C and CAL1 were unaffected by the short RNAPII inhibitor treatment, either at the total protein level or for cellular localization. However, insights into the dCENP-A loading process can be deduced from their centromeric retention after high-salt extraction. CAL1 extraction from chromatin mirrored the behavior of unstable TI-dCENP-A^HA^ in inhibitor-treated cells, as it was removed from chromatin fractions by high salt and displaced from centromeres if loosely associated proteins were extracted by salt treatment before PFA fixation (Fig. 7 A). In contrast, dCENP-C could still be detected in salt-extracted cells in which centromere clustering remained intact. This suggests that dCENP-A associates with centromeres through CAL1 but remains bound to its chaperone until transcription-mediated chromatin remodeling allows dCENP-A nucleosome incorporation.

## Materials and methods

### Cell culture

*Drosophila* S2 Schneider cells were grown at 25°C in Schneider’s *Drosophila* medium (SERVA) supplemented with 10% FCS and antibiotics (0.3 mg/ml penicillin and 0.3 mg/ml streptomycin). Cells were transfected using the XtremeGENE HP transfection reagent (Roche), and stable lines were selected with 100 µg/ml Hygromycin B and/or 2 µg/ml puromycin. Treatment lengths of drugs are described in the figure legends. Drug concentrations were 125 µM DRB (Cambridge BioScience), 10 µM tripto (Stratech Scientific), and 1 mM 4-hydroxy tamoxifen (Sigma-Aldrich).

### Cloning and DNA constructs

HA-ERT2 was amplified by PCR from pMY-ERT2-ires-GFP (D. van Essen, Institute of Research on Cancer and Aging, Nice, France) using the ERT2 primers and inserted into pMT-CID-V5-hygro (Olszak et al., 2011) using SacII–AgeI to produce pMT_dCENP-A-HA-ERT2-hygro (TI-dCENP-A^HA^). Into this construct, GFP was inserted using XhoI–SacII by amplifying GFP by PCR from pMT-CID-GFP (Olszak et al., 2011) using the GFP_nostop primers to create pMT_dCENP-A-GFP-HA-ERT2-hygro. pMT-dCENP-A-mCherry-hygro was cloned by inserting mCherry using XhoI–SacII into pMT-CID-V5-hygro. Full-length Rpb3 was cloned from cDNA using Rpb3 primers and EcoRV–NotI inserted in-frame behind an EGFP containing modified pIB/v5 vector (Invitrogen).

### IF staining

Generally, cells were settled for 20 min on polylysine-coated microscopy slides and fixed for 7 min in 3.7% formaldehyde solution (Sigma-Aldrich) in PBS or −20°C methanol. For mitotic detection of GFP-Rpb3, cells were prelysed using PBS/0.1% Triton X-100 (PBS-T) for 3 min. After a wash in PBS-T, samples were blocked with Image-iT FX signal enhancer (Invitrogen) for at least 30 min before primary antibody staining overnight. Samples were washed 3× for 10 min in PBS-T and incubated in secondary antibody for 1 h. After three further washes in PBS-T, samples were stained with DAPI for 3 min, washed with PBS-T, mounted in SlowFade (Invitrogen), and sealed with nail polish.

For fixation after salt preextraction, cells were seeded on polylysine-coated coverslips positioned in a six-well plate 1 d before treatment. The last 30 min of the 4OHT/inhibitor treatment were performed at RT in PBS-T with a final concentration of 0.5 M NaCl (all earlier treatments still present). Without disturbing the plate, PBS-T was removed and cells were fixed for 7 min with 3.7% formaldehyde solution (Sigma-Aldrich) in PBS. Antibody staining was performed as described above.

All antibody incubations were performed in a 1:1 mix of PBS-T and 10% normal goat serum (Life Technologies). Unless otherwise noted, all antibodies were used with a 1:100 dilution: chicken α dCENP-A (1:20; own antibody), rat α dCENP-A (4F8; E. Kremmer/A. Schepers, Helmholtz Zentrum München, Neuherberg, Germany), mouse α tubulin (Sigma-Aldrich), rabbit α dCENPC (1:200; C. Lehner/S. Heidmann, University of Zurich, Zurich, Switzerland), rat α HA (1:20; 3F10; Merck), mouse α GFP (Mab496; D. van Essen/S. Saccani, Institute of Research on Cancer and Aging, Nice, France), and mouse α v5 (Invitrogen). Secondary antibodies coupled to Alexa Fluor 488, 555, and 647 fluorophores (Invitrogen) were used at 1:100 dilutions. Counterstaining of DNA was performed with DAPI (5 µg/ml; 3 min).

### Chromosome spreads

0.2 × 10^5^ cells were arrested for 30 min with 1 µg/ml colcemid. Supernatant after centrifugation (3 min, 1,000 *g*) was discarded, and cells were resuspended in 500 µl of 0.5% (wt/vol) sodium citrate. After 10-min incubation, each sample was transferred into a single-chamber cytospin tunnel and spun on a polylysine-coated slide for 10 min at 900 rpm (high-acceleration) in a Shandon Cytospin 4. After the spin, slides were immediately fixed in 4% PFA, then washed twice in PBS followed by two washes in 2× SSC buffer.

### RNA/DNA-FISH

FISH analysis was performed on mitotic chromosome spreads of cells treated for 2 h with DMSO, DRB, or tripto. The SAT III probe was produced as previously described (Rošić et al., 2014), using the pCR-SAT III vector (S. Erhardt, Zentrum für Molekulare Biologie der Universität Heidelberg, Heidelberg, Germany) with SAT III forward and reverse primers in a PCR reaction with ChromaTide Alexa Fluor 488–5-dUTP nucleotides (Molecular Probes) according to the manufacturer’s instructions and used at 100 ng/reaction. An IDT DNA oligo labeled at the 3′ end with Alexa Fluor 488 was used for the TTCTC probe at 40 µM/reaction. Probes for DNA and RNA FISH were diluted in 50 µl FISH hybridization buffer (50% formamide and 10% dextran sulfate in 2× SSC) and incubated at 80°C for 5 min. For DNA-FISH, the probes were added to the slides with the cells and incubated at 80°C for 5 min before hybridization was performed at 37°C overnight. For RNA-FISH, the probes were added to the slides with the cells and incubated at 37°C overnight. Slides were washed three times in 50% formamide/2× SSC and three times in 2× SSC at 42°C and fixed in 4% PFA for 10 min.

Subsequent antibody staining was performed in the dark as described above. For RNase treatment, cross-linking was reversed by incubating the slides for 5 min at 80°C and for 40 min at 37°C in 12.5 µl pure RNase cocktail enzyme mix (AM2286). After RNase treatment, slides were once more fixed in 4% PFA for 10 min.

### Click-iT chemistry

Global RNA transcription was detected using the Click-iT RNA Alexa Fluor 488 Imaging kit (Thermo Fisher Scientific; labeling: 5 min/4 mM EU). After labeling, cells were pelleted and resuspended twice in 1 ml medium to allow unbound EU to diffuse (10 min; 400 rpm), before cells were settled for 12 min and fixed as usual. Click-iT reaction was performed according to the manufacturer’s instructions.

For qPCR of nascent RNA, we used the Click-iT Nascent RNA capture kit according to the manufacturer’s instructions. Cells were treated with DMSO, DRB, or tripto for a total of 2 h, and 0.5 mM EU was added for the last 30 min of the incubation. 10 µg EU-RNA was subsequently labeled with 0.5 mM biotin azide and 1 µg biotinylated RNA purified with 32 µl magnetic beads, and the purified RNA bound to the beads was immediately used as a template for cDNA synthesis. cDNA synthesis was performed using the SuperScript VILO cDNA synthesis kit. Triplicates of 1:20 dilutions of the cDNA were used for qPCR, which was performed using Absolute QPCR Mix, SYBR Green with ROX.

### Elutriation of Schneider cells

Elutriation experiments were performed at 4°C/on ice or at RT depending on downstream applications. 250 × 10^6^ exponentially growing Schneider S2 cells were elutriated in an Avanti J-20 XP centrifuge using a JE-5.0 rotor. Centrifugation speed was kept constant at 3,250 rpm, and cellular debris was first depleted from samples using a counterflow rate of 20 ml/min. Successive increases of the counterflow rate allowed the sampling of fractions 1 (25 ml/min), 2 (30 ml/min), 3 (35 ml/min), 4 (40 ml/min), and 5 (50 ml/min). 150 ml was collected for each fraction before cells were concentrated again (25 min at 1,000 *g*) and directly settled on polylysine-coated slides (4°C/on ice) or submitted to nascent RNA labeling (RT).

### Microscopy and image analysis

All images were taken at RT on a DeltaVision Elite Imaging System and were deconvolved and quick projected (maximum intensity) using softWoRx Explorer Suite 2.0 (Applied Precision). Images of fixed cells were taken with a CoolSnapHQ camera and 50–65 *z*-stacks at 0.2-µM increments using an Olympus UPLAN S-APO 100× oil, 1.4-NA objective. Time-lapse imaging was performed with a Cascade2_1K EMCCD camera using a PLAN-APO 60× oil, 1.4-NA objective and a time lapse of 10 min. 25 *z*-stacks at 0.4-µM increments were taken for dCENP-A imaging shown in Fig. S2 D, and 10 *z*-stacks at 1-µM increments were used for quantification shown in Fig. S3 F. Quantification of signal intensities was performed using softWoRx Explorer Suite or ImageJ. 5–10 random pictures were taken for the analysis, with the same exposure conditions for all treatment types. The mean background of noncentromeric nuclear measurements was subtracted from the measured centromeric signal. P-values were determined using Kolmogorov–Smirnov test or Student’s *t* test.

### Whole-cell lysates and cellular fractionation

All steps were performed on ice/at 4°C and all used buffers containing in addition protease inhibitor cocktail tablets (cOmplete Cocktail Tablets; Roche) and 0.5 mM PMSF. Cells were washed twice with PBS before lysis. For whole-cell lysates, cell pellets were resuspended in buffer L (50 mM Tris-HCl, 150 mM NaCl, 1 mM EDTA, 1% Triton X-100, and 1 mM MgCl_2_) and sonicated 10× at intervals of 30 s on medium (Bioruptor300; Diagenode). For the preparation of RNAPIISer2p/Ser5p, PhosSTOP Easypack (Roche) was added to the buffer. For cellular fractionation, samples were incubated for 5–10 min in lysis buffer (20 mM Hepes, 3 mM MgCl_2_, 0.1% Triton X-100, and 1 mM DTT), homogenized by 25 strokes with a 23G needle, and incubated for a further 10 min. Nuclei were pelleted (5 min/2,000 *g*), and the supernatant served as the cytoplasmic fraction. The pellet was washed once, resuspended in extraction buffer (20 mM Hepes, 10% glycerol, 80 or 500 mM NaCl, 1 mM MgCl_2_, 0.1% Triton X-100, and 1 mM DTT) and rotated overnight. Supernatant after centrifugation (5 min/maximum speed) served as the nucleoplasmic fraction. The chromatin pellet was washed once, resuspended in extraction buffer (containing only 0.15 M NaCl) plus Benzonase (Novagen; 100 U/ml), and rotated for 1 h. Supernatant after 5 min/maximum speed served as the chromatin fraction.

### Chromatin preparation and MNase digest

Nuclei from 120 × 10^6^ cells for each treatment condition were prepared as described before (Gilbert et al., 2003) with an NP-40 concentration of 0.5% in buffer B. For MNase digest, the nuclei concentration was adjusted to 20 A260 in nuclei buffer R (85 mM KCL, 5.5% sucrose, 10 mM Tris, pH 7.5, 1 mM CaCl_2_, 1 mM MgCl_2_, and 250 µM PMSF) and digested with 8 U MNase (Thermo Fisher Scientific) for 8 min at RT. Digestion was stopped by addition of EDTA to 10 mM, the sample was spun down, and chromatin was released overnight at 4°C in TEEP_20_ (10 mM Tris, pH 8, 1 mM EDTA, 1 mM EGTA, 250 µM PMSF, and 20 mM NaCl) containing 300 ng/ml l-α-lysophosphatidylcholine (Sigma-Aldrich). Nuclear debris was removed through centrifugation, and the soluble chromatin was divided into two samples (one brought to 650 mM NaCl) before fractionation on sucrose step gradients.

### Sucrose gradient sedimentation

Soluble chromatin was fractionated on 10–50% (wt/vol) isokinetic sucrose gradients containing TEEP_80_ (as TEEP_20_ with 80 mM NaCl) or TEEP_650_ (as TEEP_20_ with 650 mM NaCl) by centrifugation (135 min at 41,000 rpm) in a Beckman SW41 rotor. Gradients were displaced upward with continuous monitoring of the UV absorbance profile. Fractions were collected for 30 s per sample.

### Western blot analysis

Samples were boiled for 10 min in loading buffer separated on 10–12% (fractionation assay) or 6% (RNAPII whole-cell lysates) SDS-PAGE gels and processed for Western blotting using mouse α HA (1:10,000; 12CA5), rabbit α dCENP-A (1:2,000; Active Motif), mouse α tubulin AA4.3 (1:1,000; Developmental Studies Hybridoma Blank), rat α RNAPIISer2p (1:500), and rat α RNAPIISer5p (1:500; E. Kremmer/A. Schepers, Helmholtz Zentrum München, Neuherberg, Germany). Secondary antibodies coupled to HRP (Dianova) were used at 1:10,000.

### Cell cycle analysis

10^6^ cells were pelleted in a FACS tube (7 min; 1,000 *g*) and fixed at 4°C overnight in 70% ethanol. Fixed cells were stained for 1 h in the dark (50 µg/ml propidium iodide and 100 µg/ml RNase in PBS) and directly subjected to analysis on a BD FACScalibur Flow Cytometer using a gate for single cells.

### Online supplemental material

Fig. S1 (A–C) shows the centromeric localization of GFP-Rpb3 in anaphase and telophase and in midbody-containing early G1 cells and the intensities for centromeric Rpb3-GFP signal in both interphase and mitotic cells. Fig. S1 (D and E) depicts control stainings for nascent RNA transcript production in S-phase cells and without EU incubation. Fig. S2 shows that chromatin/centromeric localization of TI-dCENP-A^HA^ depends on 4OHT and is similar to the incorporation of dCENP-A^mCherry^. Fig. S3 demonstrates the effectiveness of the inhibitor treatment, the effect on cell cycle progression, and the reduction in marker gene expression of various polymerase complexes (note that in the absence of RNAPII transcripts in the DRB sample, RNAPI and RNAPIII transcripts are overrepresented). Fig. S4 proves the stability of released TI-dCENP-A^HA^ in cells largely destroyed by salt extraction and shows the behavior of released TI-dCENP-A^HA^ in methanol fixation and the effect of transcriptional inhibition on the protein levels of dCENP-C and CAL1. Table S1 lists the qPCR primers used in Fig. S3 E, primers for FISH probe production used in Fig. 5, and primer for cloning of DNA constructs.

## Supplementary Material

Supplemental Materials (PDF)
