# Supplementary material for: Centromere transcription allows CENP-A to transit from chromatin association to stable incorporation
Source: J Cell Biol. 2018 Jun 4;217(6):1957–72. doi: 10.1083/jcb.201611087 (PMC5987708; doi:10.1083/jcb.201611087)
Supplement: Supplemental Materials (PDF) [file JCB_201611087_sm.pdf]

Supplemental material

Bobkov et al., https://doi.org/10.1083/jcb.201611087

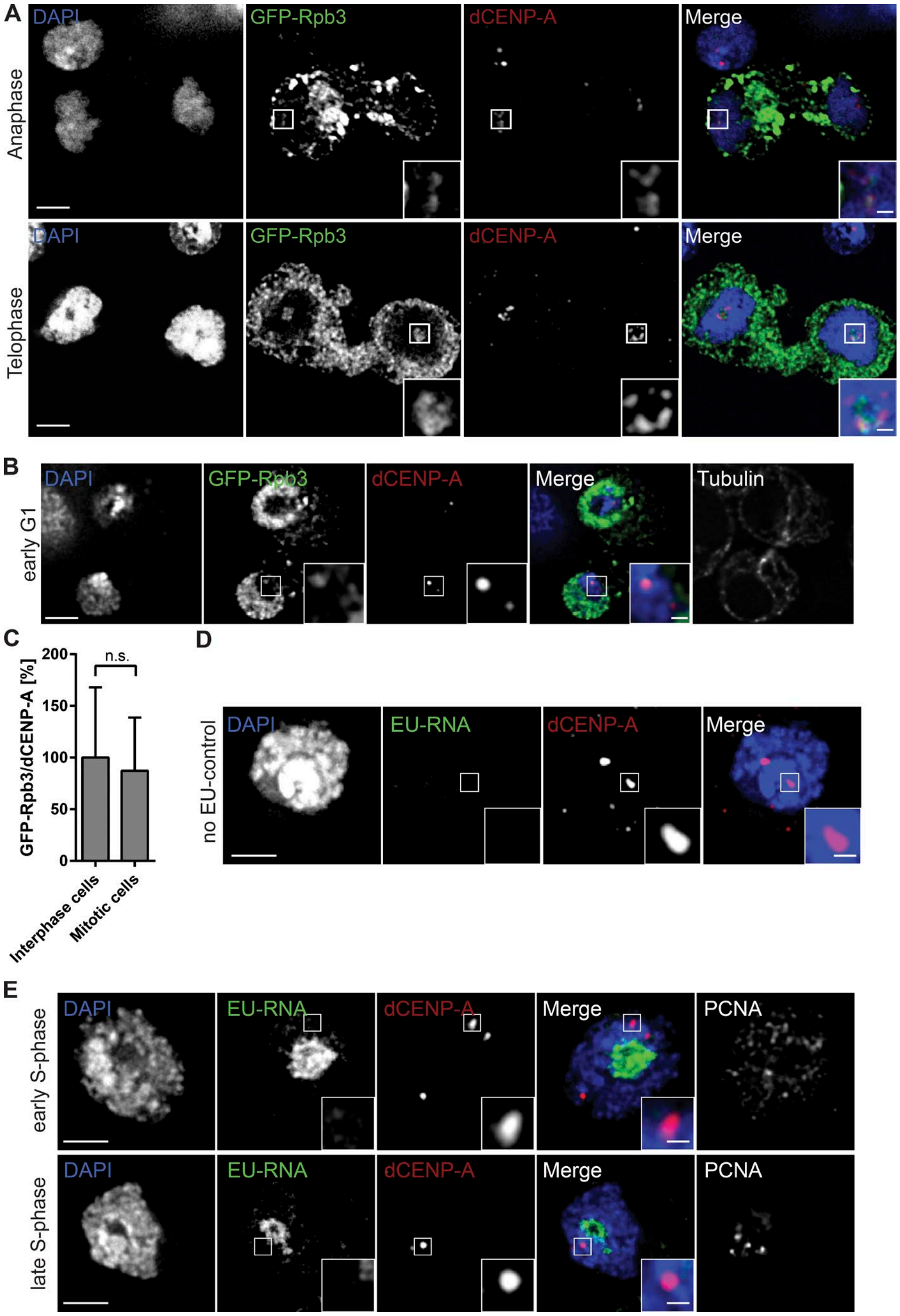

Figure S1. **RNAPII and centromere-associated transcripts are present at centromeres in late mitosis and early G1. (A)** Single optical section of fixed anaphase and telophase S2 cells expressing GFP-Rpb3 and immunostained for dCENP-A. Bar, 3 µm. Boxes indicate the 3× enlarged inset (bar, 0.5 µm). **(B)** Single optical section of early G1 cell expressing GFP-Rpb3. Centromeres are marked by dCENP-A and the midbody by tubulin immunodetection. Bar, 3 µm. Boxes indicate the 3× enlarged inset (bar, 0.5 µm). **(C)** Signal intensities of centromeric GFP-Rpb3 in interphase/mitotic cells with GFP-positive centromeres. As centromeres cluster in *Drosophila* during mitosis, measured signals were normalized to corresponding dCENP-A signals. n = 2 replicates; n = 5–15. The p-value was determined using the Student's t test. **(D)** Single optical section of fixed S2 cell on which click-iT labeling was performed without EU treatment of the cell. Scaling for EU signal is exactly the same as in Fig. 2 B. Bar, 3 µm. Boxes indicate the 3× enlarged inset (bar, 0.5 µm). **(E)** Single optical section of fixed S2 cells with nascent RNA production labeled by EU incorporation. PCNA staining served as a marker for S-phase, early S-phase (upper panel), and late S-phase (lower panel). Bar, 3 µm. Boxes indicate the 3× enlarged inset (bar, 0.5 µm).

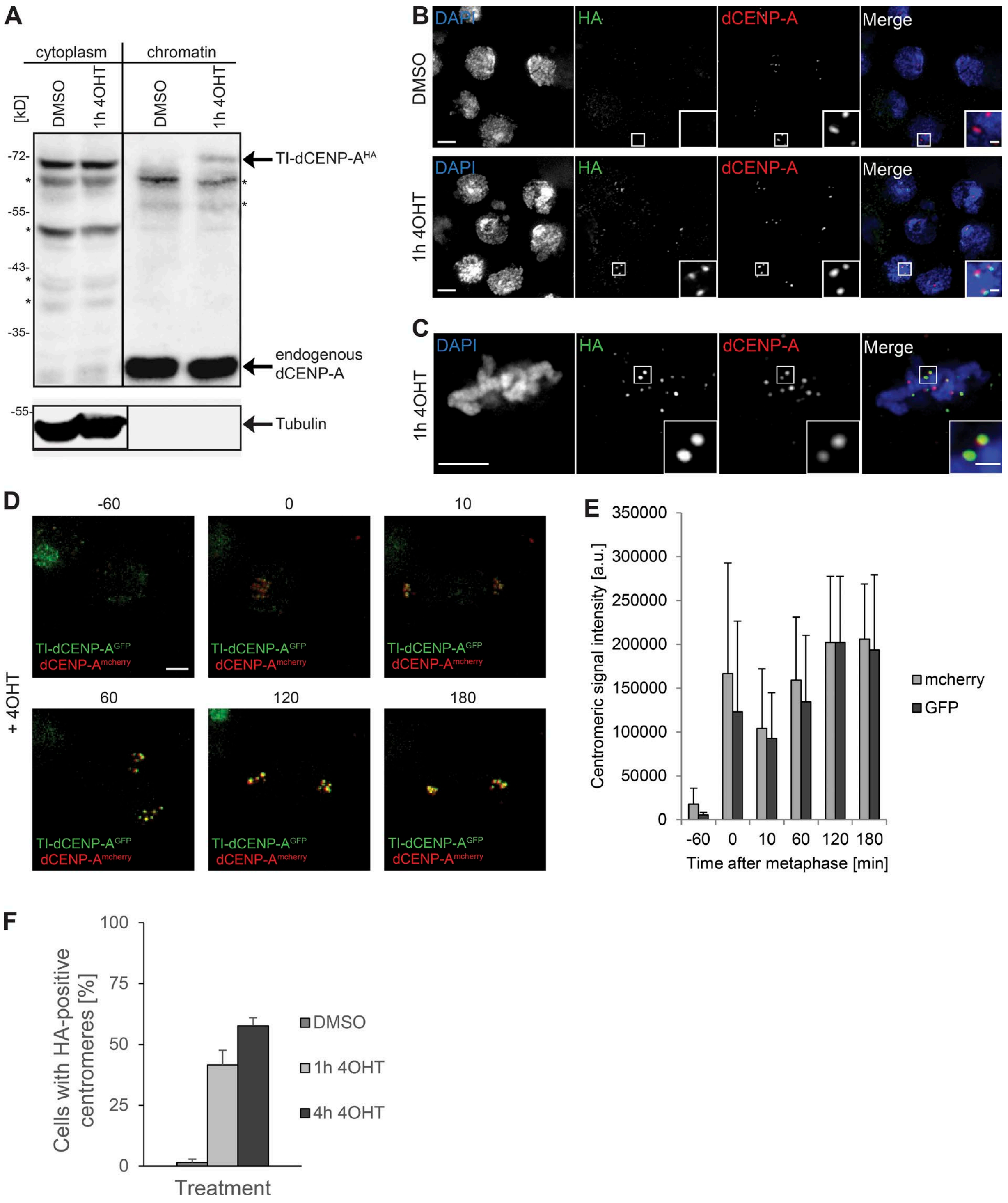

Figure S2. **A dCENP-A loading system independent of acute transcription.** **(A)** Western blot analysis showing the incorporation of TI-dCENP-A^{HA} into the chromatin fraction after addition of 4OHT. Arrows mark protein of interest, and asterisks (*) mark unspecific bands or potential degradation products. **(B)** Maximum-intensity projection of IF images of fixed S2 cells stably expressing TI-dCENP-A^{HA} and stained for HA and dCENP-A. Control cells (upper panel) and 4OHT-treated cells (lower panel) are shown. Bars, 3 µm. Boxes indicate the 3× enlarged inset (bars, 0.5 µm). **(C)** Maximum-intensity projection of fixed metaphase S2 cell stably expressing TI-dCENP-A^{HA} and stained for HA and total dCENP-A. Bar, 3 µm. Boxes indicate the 3× enlarged inset (bar, 0.5 µm). **(D)** Live-cell imaging of cells stably expressing TI-dCENP-A^{GFP} and pictured before the first incorporation of transiently expressed dCENP-A^{mCherry}. Cells were treated with 4OHT to trigger the release of TI-dCENP-A^{GFP}. Numbers above pictures indicate minutes before/after metaphase. Bar, 3 µm. **(E)** Quantification of cells exemplified in D. *n* = 2 replicates; *n* = 5 cells; data are mean + SD. **(F)** Quantification of the amount of cells that respond to 4OHT treatment with localization of TI-dCENP-A^{HA} to centromeres. *n* = 2 replicates; *n* = 100–200 cells; data are mean + SD.

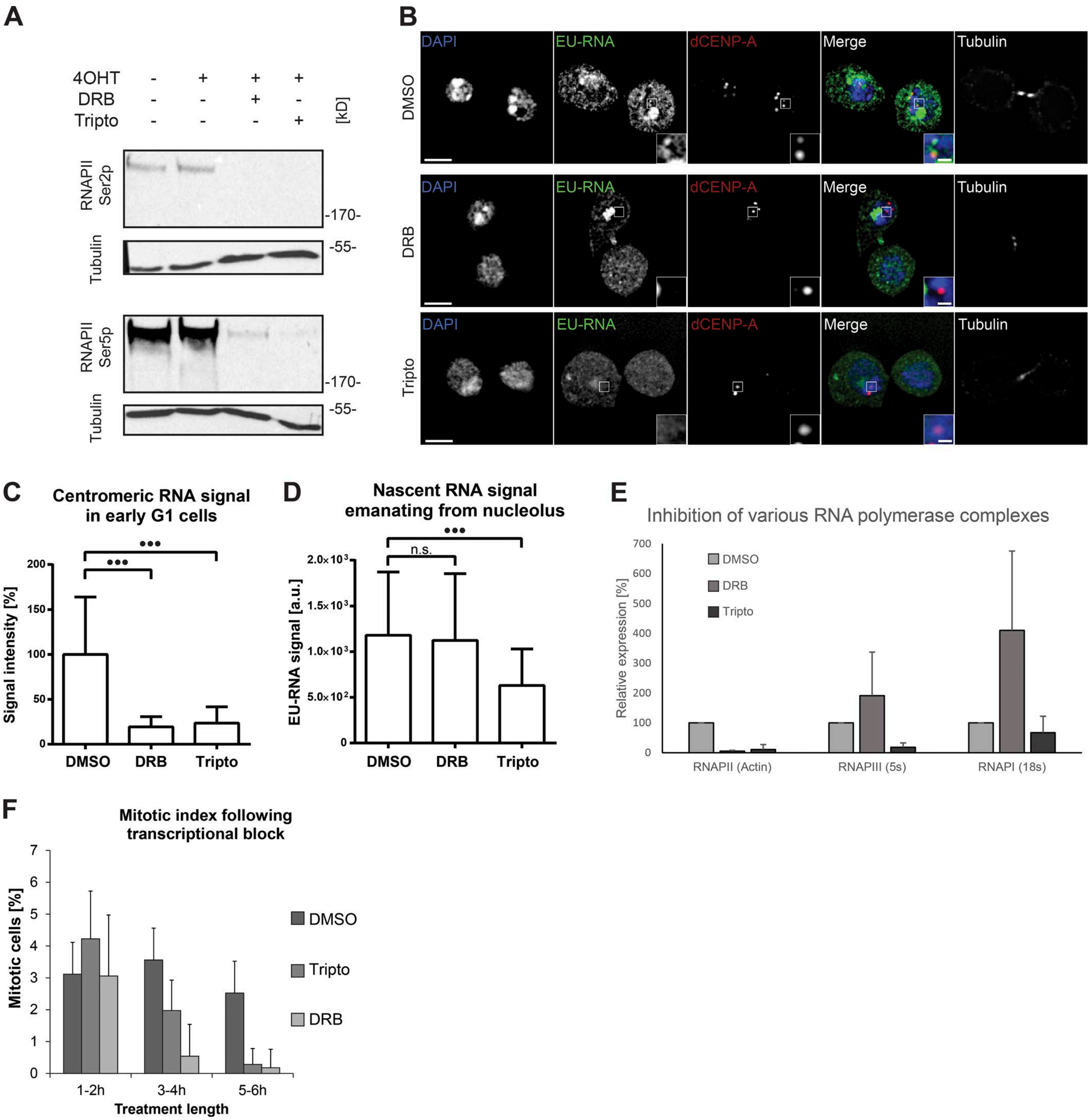

Figure S3. **Nascent centromeric transcripts are produced by RNAPII. (A)** Western blot demonstrating the reduction of phosphorylation of RNAPIISer2 and RNAPIISer5 by the inhibitor treatment. **(B)** Single optical section of early G1 cells with nascent RNA production labeled by EU incorporation. Centromeres are marked by dCENP-A and the midbody by tubulin immunodetection. Control and inhibitor treated cells are shown. Bars, 3 µm. Boxes indicate the 3× enlarged inset (bars, 0.5 µm). **(C and D)** Quantification of centromeric RNA signals (C) and nucleolar RNA signals (D) in cells exemplified in B. *n* = 3 replicates; *n* = 10 cells; data are mean + SD. The p-value was determined using Student's *t* test. •••, P ≤ 0.001. **(E)** qPCR showing the relative expression of marker genes for RNAPI–III in control and inhibitor-treated cells. Note that in the absence of RNAPII transcripts in the DRB sample, RNAPI and RNAPIII transcripts are highly overrepresented. *n* = 3 replicates. Data are normalized to expression in control treated cells and represented as mean + SD. **(F)** Quantification of the amount of cells that enter mitosis upon transcriptional inhibition. No mitotic arrest is induced by the inhibitor treatment. *n* = 3 replicates, *n* = 400–750 cells; data are mean + SD.

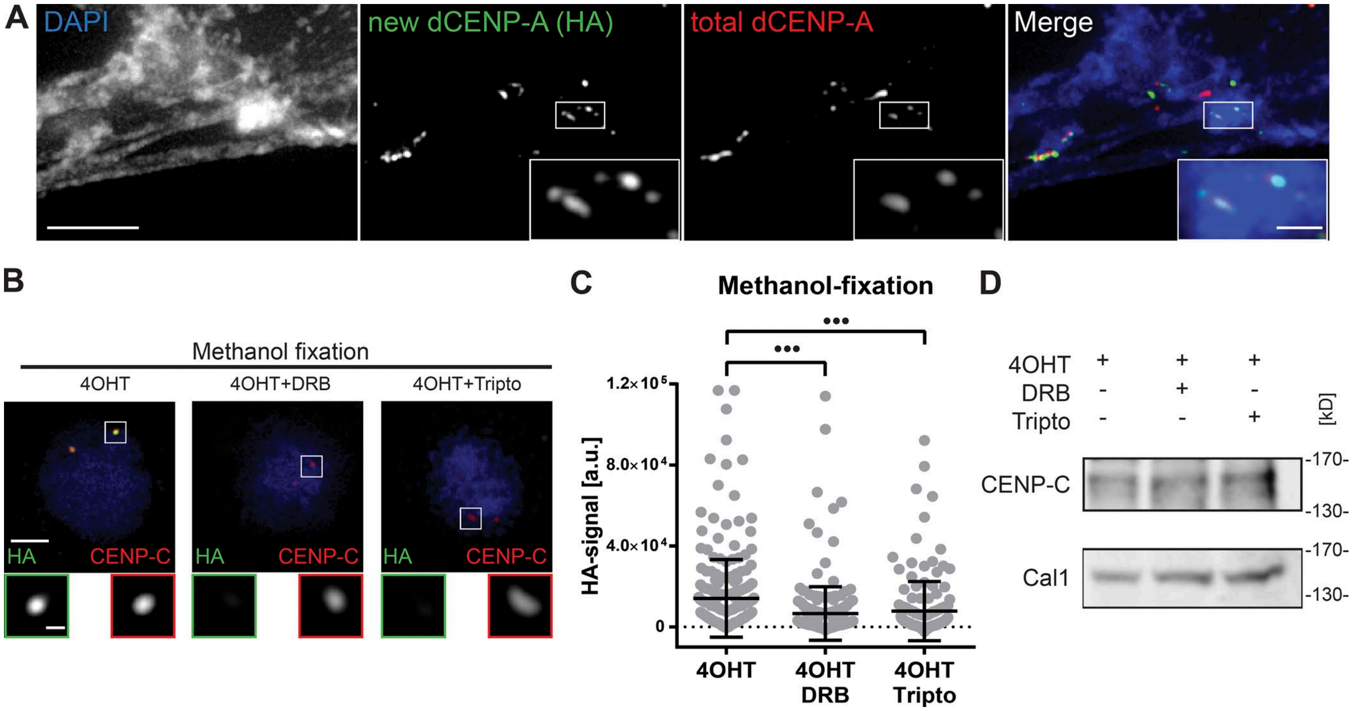

Figure S4. **Transcriptional inhibition prevents stabilization of new dCENP-A. (A)** Maximum-intensity projection displaying the retention of new dCENP-A in cells with least preserved nuclear integrity. dCENP-A staining served as a marker for centromeres. Bar, 3 µm. Boxes indicate the 3× enlarged inset (bar, 0.5 µm). **(B)** Maximum-intensity projection of cell stably expressing TI-dCENP-A^{HA} fixed in methanol. Respective treatments are indicated above each picture. Bar, 3 µm. 3× magnification of boxed area is shown below (TI-dCENP-A^{HA} [green] and dCENP-C [red]; bar, 0.5 µm). **(C)** Quantification of pictures exemplified in B. *n* = 3 replicates, *n* = 30–75 cells; data are mean ± SD. The p-value was determined using Kolmogorov–Smirnov test. •••, P ≤ 0.001. **(D)** Western blot analysis showing that CENP-C and CAL1 protein levels are not altered by the inhibitor treatment.

Table S1. **Primer sequences**

Name	Forward primer (5′–3′)	Reverse primer (5′–3′)
5S rDNA	AAACTGTGCGTCATCGTGTG	TGGACTGCGATATGCGTAAA
Actin	TCGCCATCTAACCGACTACC	AGTGCGGTGATTTCCTTTT
18S rDNA	AGCCTGAGAAACGGCTACCA	AGCTGGGAGTGGGTAATTTACG
SAT III	TATTCTTACATCTATGTGACC	GTTTTGAGCAGCTAATTACC
GFP_nostop	ATTCTCGAGCATGGTGAGCAAGGGCGAGGA	ATTCCGCGGGGCGGCGGTCACGAACTC
ERT2	AAAGGATCCAGCCCGCGGAGCTATCCATACGATGTGCCGGAT TACGCTGGCGATATGTCTGCTGGAGACATGAGAGCTGCCAA	TTTACCGGTTTAAGCTGTGGCAGGGAAACCCTCTGCCTCCCCCGTG
Rpb3	ATAGATATCCAAACCGCAATGCCGTACGCCAACC	AATGCGGCCGCTATGTAAATCAAACGGCCAATGC

rDNA, ribosomal DNA.
